# Genome sequence of *Flavobacterium* sp. MAHUQ-57 isolated from the soil sample of a rice field located in Dighalgram, Magura, Bangladesh

**DOI:** 10.1128/mra.00953-25

**Published:** 2026-01-13

**Authors:** Md. Amdadul Huq, M. Mizanur Rahman, Md. Shahedur Rahman

**Affiliations:** 1 Department of Life Sciences, College of BioNano Technology, Gachon University, Seongnam, Republic of Korea; 2 Department of Biotechnology and Genetic Engineering, Faculty of Biological Science, Islamic University, Kushtia, Bangladesh; 3 Department of Genetic Engineering and Biotechnology, Jashore University of Science and Technology, Jashore, Bangladesh; Queens College Department of Biology, Queens, New York, USA

**Keywords:** genome sequence, *Flavobacterium *sp. MAHUQ-57, rice field

## Abstract

This study reports the draft genome sequence of *Flavobacterium* sp. MAHUQ-57, a strain isolated from the soil sample of a rice field located in Dighalgram, Magura, Bangladesh, in 2019. The genome consists of a 3,472,804 base pair chromosome assembled into 23 contigs, encoding 3,202 predicted protein-coding genes.

## ANNOUNCEMENT

*Flavobacterium* is a genus of gram-negative, nonmotile and motile, rod-shaped bacteria that consists of 330 validly published species (https://lpsn.dsmz.de/genus/flavobacterium). Members of the genus *Flavobacterium* were isolated from different habitats, such as plants, animals, algae, seawater, freshwater, soil, rhizosphere, compost, sediments, lagoons, Antarctic lakes, glaciers, activated sludge, water reservoirs, and wastewater treatment plant (1–4). During investigations on the biodiversity of bacteria in the rice field, a strain of genus *Flavobacterium*, designated as *Flavobacterium* sp. MAHUQ-57 was isolated, and its draft genome assembly is presented in this study.

Strain MAHUQ-57 was isolated from the soil sample of a rice field, located in Dighalgram, Magura, Bangladesh (GPS map: 23.410203, 89.332807). Soil samples were collected from the rhizosphere surrounding the plant roots. One gram of soil sample was suspended in 9 mL of sterile 0.85% (wt/vol) NaCl solution. The suspension was serially diluted up to a 10^−6^ dilution, and 100 µL of individual dilution was spread onto R2A agar plates. Then, the plates were placed at a 30°C incubator for 3 days. Single colonies were purified by repeated streaking on fresh R2A agar plates. The genomic DNA was extracted from the bacterial culture that was grown in R2A broth for 24 h using Solg Genomic DNA Prep kit (Solgent, Republic of Korea), according to the manufacturer’s protocol. DNA was fragmented using a tagmentation enzyme (Nextera XT kit, Illumina, San Diego, USA). The DNA fragments were size selected using AMPure XP beads to isolate those within the desired range, typically 200–300 base pairs (bp) for standard Illumina sequencing libraries. The library for sequencing was prepared using the Nextera XT DNA Library Prep Kit (Illumina). After library preparation, sequencing was carried out using the Illumina HiSeq 3000 platform with paired-end 2 × 150 bp reads following the standard protocol provided by the manufacturer. Illumina’s bcl2fastq software version 2.20.0 was utilized with default parameters to demultiplex the raw reads and convert them into FASTQ format. Reads were quality-controlled using FastQC (5), and adapter sequences and low-quality bases were trimmed with Trimmomatic version 0.38 (6). The genome sequence was assembled by SOAPdenovo v2.04 assembler (7). The genome was annotated with the NCBI Prokaryotic Genome Annotation Pipeline version 6.3 (8–10). The completeness and contamination were assessed by CheckM tool, version 1.2.4 (11). The strain was identified using a whole genome-based taxonomic analysis in the Type (Strain) Genome Server (https://tygs.dsmz.de). The genome-to-genome distance bioinformatics tool (12) was used to measure *in silico* DNA-DNA hybridization values. The best matches to the strain MAHUQ-57 genome are the type strain *Flavobacterium amniphilum* KYPY10 (accession NZ_JAMLJL000000000) with *in silico* DNA-DNA hybridization values of 22.4%.

The number of reads generated by genome sequencing was 3,072,309. The genome of *Flavobacterium* sp. MAHUQ-57 is 3,472,804 bp long with a GC content of 35.5%. The assembly contains 23 contigs with a coverage of 139×. A total of 3,262 genes were predicted, of which 3,202 are protein-coding genes. The details of genomic features are presented in Table 1.

**TABLE 1:** Genomic features of *Flavobacterium* sp. MAHUQ-57

Feature	Result
Description of source	
Location	Dighalgram, Magura, Bangladesh
Time	2019
Type	Soil of rice field
Sequencing summary	
Coverage	139×
Total bases	3,472,804
GC content	35.5%
Assembly report	
Contigs	23
Contig *L*_50_	3
Contig *N*_50_	653.8 kb
Genome length	3.5 Mb
Annotation report	
Genes (total)	3,262
CDSs (total)	3,209
CDSs (with protein)	3,202
ncRNAs	3
tRNA	44
Quality analysis	
Completeness	99.22%
Contamination	0.17%

## Data Availability

The draft genome sequence of *Flavobacterium* sp. MAHUQ-57 was deposited in GenBank under the accession number JBPZTE000000000. The raw reads are available in SRA under the accession number SRR33604967. The version described in this paper is the first version, JBPZTE000000000.1. The data have been deposited in GenBank under the BioSample number SAMN50309237 and the BioProject number PRJNA1299606. The genome assembly is available under the accession number GCA_051908335.1.
